# Neuroinflammation and neuroprogression produced by oxidative stress in euthymic bipolar patients with different onset disease times

**DOI:** 10.1038/s41598-022-21170-y

**Published:** 2022-10-06

**Authors:** Daniela Delwing-de Lima, Luiz Arthur Rangel Cyrino, Gabriela Kozuchovski Ferreira, Débora Delwing Dal Magro, Claudia Regina Calegari, Heloisi Cabral, Natalia Cavichioli, Silvia Aparecida Ramos, Oliver Matheus Ullmann, Yasmin Mayer, Luana Carla Pscheidt, Maria Augusta Schramm, Maria Cecília Tomasi, Felipe Luis Schmoller Stammerjohann, Larissa Delmonego, Maria Helena Packer, Heloiza Fiamoncini

**Affiliations:** 1 Programa de Pós-Graduação Em Saúde E Meio Ambiente, Universidade da Região de Joinville– UNIVILLE, Rua Paulo Malschitzki, 10 - Zona Industrial Norte, Joinville, SC CEP 89201-972 Brasil; 2 Departamento de Medicina, Universidade da Região de Joinville UNIVILLE, Rua Paulo Malschitzki, 10 - Zona Industrial Norte, Joinville, SC CEP 89201-972 Brasil; 3 Departamento de Farmácia, Universidade da Região de Joinville UNIVILLE, Rua Paulo Malschitzki, 10 - Zona Industrial Norte, Joinville, Joinville, SC CEP 89201-972 Brasil; 4 Departamento de Psicologia, Universidade da Região de Joinville UNIVILLE, Rua Paulo Malschitzki, 10 - Zona Industrial Norte, Joinville, Joinville, SC CEP 89201-972 Brasil; 5 Programa de Pós-Graduação Em Ciência E Engenharia de Materiais, Universidade Sociedade Educacional de Santa Catarina UNISOCIESC, Joinville, Santa Catarina Brasil; 6 Departamento de Ciências Naturais, Centro de Ciências Exatas E Naturais, Universidade Regional de Blumenau, Rua Antônio da Veiga, 140, Blumenau, SC CEP 89012-900 Brasil; 7 Departamento de Psicologia, Universidad Europea del Atlántico, UNEATLANTICO, Calle Isabel Torres, 21, Santander, Spain; 8 Departamento de Ciências Biológicas, Universidade da Região de Joinville UNIVILLE, Rua Paulo Malschitzki, 10 – Zona Industrial Norte, Joinville, SC CEP CEP 89201-972 Brasil; 9 Departamento de Biomedicina, Centro de Ciências da Saúde, Universidade Regional de Blumenau, Rua Antônio da Veiga, 140, Blumenau, SC CEP 89012-900 Brasil

**Keywords:** Neuroscience, Biomarkers

## Abstract

Bipolar disorder (BD) is associated with systemic toxicity, represented by changes in biomarkers associated with mood episodes, leading to neurological damage, which may reflect cognitive functions and functionality and the progression of the disease. We aimed to analyze the effect of four biomarkers, superoxide dismutase (SOD), catalase (CAT), glutathione peroxidase (GSH-Px), and thiobarbituric acid reactive substances (TBA-RS), related to oxidative stress in BD and to correlate them with cognitive functions and functionality. We studied 50 bipolar types I/II patients in the euthymic phase, which was divided into two subgroups with 25 patients each (≤ 3 years and ≥ 10 years of diagnosis, from the first episode of mania) and 25 control patients. To analyze frontal cognitive functions and functionality, we used the Frontal Assessment Battery (FAB) and Functioning Assessment Short Test (FAST) tests, respectively. The scores of the FAST and FAB tests showed an increase and decrease respectively, in both bipolar groups, when compared to the control group, demonstrating impairment in cognitive functions and functionality since the disease onset. In addition, changes occurred in all six domains of the FAST test, and in four domains of the FAB test in bipolar patients when compared to the control group. Regarding oxidative stress biomarkers, we did not find changes in SOD and GSH-Px activities; however, a significant increase in CAT activity and lipid peroxidation was observed in both groups, although the patients were euthymic and medicated. These results allow us to raise the hypothesis that since the beginning of the disease, the euthymic bipolar patient has presented a level of oxidative stress, which gets worse with the evolution of the disease, promoting impairments in the frontal cognitive functions and functionality gradually.

## Introduction

### Bipolar disorder, neuroinflammation and neuroprogression

Bipolar disorder (BD) is a chronic and severe disease associated with a high rate of clinical comorbidities. This pathology presents chronic and recurrent mood changes, characterized by cyclic episodes of depression and mania, which can be interspersed with periods of mood stability (euthymia), remission of symptoms, and returning the patient to a stable mood. The diagnosis is made based on the manifestation of at least one manic or hypomanic episode during life, with the presence of a manic episode confirming the diagnosis of type I BD, while the presence of a hypomanic episode confirms the diagnosis of type II BD, which are the two main diagnostic subtypes of BD^1^. Recent studies have shown that from multiple mood episodes, neurological changes occur within neurotransmission, neuroplasticity, growth factor signaling, and metabolism, as well as oxidative stress and neuronal apoptosis altering brain development and leading to neuroinflammation^2^. All these neuronal abnormalities in BD can result in gross morphological changes, such as reduced prefrontal and hippocampal volumes leading to a reorganization of brain circuits, resulting in cognitive, emotional, and functional deficits^2^. Other studies have shown that the severity of BD and loss of response to treatment are correlated with the number of previous episodes^3^. Thus, the progressive structural and biochemical changes in the prodromal and early stages of the disease will evolve to more advanced stages^4^, producing a slow evolution of the clinical process, called neuroprogression. Thus, the typical bipolar patient exhibits a gradual decline in behavior and cognitive functions, such as; attention, memory, language, visual, spatial abilities and executive functions), with impairments in functionality and with a weaker response to treatment, leading to higher rates of clinical comorbidities and an increased risk of suicide. In this study, we evaluated some executive functions such as (working memory, flexible thinking, inhibitory control among others), using a frontal assessment test. This slow progression prevents early diagnosis and consequently the rapid initiation of appropriate treatment. Thus, both the diagnosis and the therapeutic regimen can take several years, resulting in substantial clinical, cognitive and functional impairment^5,6^. In this context, with the progression of the disease, there is an increase in the frequency and severity of episodes of mania and depression over the years, leading to an increase in the number of associated medical and psychiatric comorbidities^7^, with an imbalance between pro- and anti-inflammatory factors^8^, showing a reduction in neurotrophins^9^, and increased oxidative stress^10^. Thus, BD can be seen as a multisystem inflammatory disease and is represented by changes in serum biomarkers^11^. Within this context, BD is associated with dysfunctional mitochondria, leading to an important metabolic disturbance in neurons and glial cells, which demand much energy^12^. Mitochondrial dysfunction involving disruption of the electron transport chain (ETC) is considered the main cause of chronic oxidative stress in BD, resulting in damage to the cell membrane and DNA, which further aggravates oxidative stress. These factors create a perpetuating pathogenic cycle, contributing to a chronic neuroinflammation process. Thus, the term neuroprogression is used to denote the progressive changes that BD presents from the initial stages to the more advanced stages of the disease, relating the severity of BD to the loss of response to treatment and the number of previous episodes. Several studies have shown that neurons and other brain cells suffer gradual damage from the first most intense episode of BD, and more recently, some studies seem to demonstrate that these changes can appear even in the prodromal period of the disease^4,13^. Furthermore, this research intends to corroborate the neuroprogression hypothesis, which was developed and described by Berk (2009)^5^ and Kapczinski et al. (2008)^14,15^. It seeks to understand how a disease that initially manifests with a relatively benign condition can deteriorate in a few years, producing a decreased capacity for reasoning, planning and learning and consistently altering moods might present a reduction in cognitive and functional recovery capacity to the point of preventing a bipolar patient from leading a normal life.

### Biomarkers related to oxidative stress

In the last decade, several studies have evaluated whether inflammatory processes and immune neural interactions might be involved in the pathophysiology of major depression and BD. Different groups of biomarkers were studied simultaneously from a set of targets related to oxidative stress, neurotrophins, inflammatory mediators, and energy metabolism, which were linked to the changes in BD^16^.

Among the biomarkers related to oxidative stress, there are the following substances: superoxide dismutase (SOD), catalase (CAT), and glutathione peroxidase (GSH-Px), which belong to a crucial group of antioxidant substances and act as a defense mechanism against free radicals^17^. Free radicals, whose unpaired electrons in the outermost shell are centered on oxygen (O_2_) or nitrogen atoms, are called ROS (reactive oxygen species) and RNS (reactive nitrogen species). ROS and RNS are constantly formed, and when in excess, they can cause the oxidation of biological molecules, such as proteins, DNA, and lipids (lipoperoxidation). The levels of thiobarbituric acid reactive substances (TBA-RS) in the plasma are related to lipoperoxidation. The imbalance between oxidative challenge (free radicals) and the body's antioxidant defense capacity is called oxidative stress. The set of substances that are called ROS are composed of the following elements: superoxide (O_2_•-), hydroxyl (OH•), hydrogen peroxide (H_2_O_2_), nitric oxide (NO•), nitrogen dioxide (NO_2_), and others, where most of them are formed through enzymatic and nonenzymatic reactions^18,19^. Therefore, the objective of this research was to evaluate four biomarkers of oxidative stress (SOD, GSH-Px, CAT, and TBA-RS) measured in peripheral blood samples from 50 bipolar types I/II patients who were in the euthymic phase for more than six months and compare them with 25 health control patients. Since the diagnosis of their first manic episode, we divided them into two subgroups (≤ 3 years and ≥ 10 years of the disease) and correlated them with cognitive functions and functionality using the FAB and FAST tests, respectively.

## Materials and methods

### Ethics

This study was approved by the Research Ethics Committee of Universidade da Região de Joinville—UNIVILLE (protocol number 655.037) and followed the ethical rules of the Helsinki Declaration of 1975. All participants provided written informed consent before entering the study. Each patient underwent a clinical and psychiatric evaluation, where demographic, anthropometric, pharmacological data and clinical variables were collected.

### Participants

The study evaluated 50 outpatients with BD types I/II in euthymia. The participants were divided into three distinct groups, each with 25 individuals: 25 euthymic BD patients in the early stage of disease (≤ 3 years since the diagnosis of BD from the first manic episode); 25 euthymic BD patients in the late stage of disease (≥ 10 years since the diagnosis of BD from the first manic episode); and 25 healthy controls. The bipolar patients were recruited from the Porto Seguro Psychiatric Hospital, located in the city of Curitiba, Brazil; and the healthy controls group was selected among hospital staff. The psychiatric diagnosis of BD patients for types I/II was defined in the Manual Diagnosis and Statistics of Mental Disorders (DSM-V) and confirmed by a semi-Structured Clinical Interview, according to DSM-V (SCID-5-CV). Manic and depressive symptoms were assessed using the Young Mania Rating Scale (YMRS)^20^ and the 17-item version of the Hamilton Depression Rating Scale (HAMD-17)^21^, respectively. With the HAMD-17 scale, depressive symptoms that had occurred within the last week were evaluated, and in the YMRS, manic symptoms that had presented themselves within the last 48 h were evaluated. The cutoff scores used in the study were YMRS > 7 as indicative of mania and HAMD-17 > 7 as indicative of depression^22^. The choice of time of the disease (≤ 3 years and ≥ 10 years from the first manic episode) was a consensus of the research group.

### Criteria

The inclusion criteria of bipolar patients in the euthymic stage were as follows: (a) the patients had been in the euthymic phase for at least six months; (b) active age (18—60 years); (c) no history of neurodegenerative diseases, cancer, morbid obesity or trauma; (d) none of the patients had a history of addiction or substance abuse in the last year; (e) not pregnant or breastfeeding; (f) nonsmokers; (g) patients had no significant comorbid medical conditions and did not receive medication in addition to those prescribed for their psychiatric condition; these patients should have been used for at least four weeks; and (h) patients were able to understand the procedures and protocol and provided written informed consent and did not present cognitive impairment with disability or dementia or physical disabilities, e.g., visual or hearing impairments.

### Demographic, clinical, and pharmacological data

The demographic variables of the healthy control and the euthymic patients groups, were matched by age, gender, profession, marital status, and educational level and years of education. Clinical variables evaluated were the age at onset, hospitalization and the duration of hospitalizations, illness duration (years), suicide attempts, and relatives’ antecedents of mental diseases and medications.

### Neuropsychological assessment

More recently, two tests have been used to assess the frontal cognitive functions (executive functions) and functionality of bipolar patients: FAST and FAB tests, respectively. Both tests are short tests (10 min) and composed of six domains each^23,24^.

Although initially, the FAB test was validated in patients with neurodegenerative diseases, vascular damage, and dementias^24,25^. More recently, several authors have started to research the use of the FAB test for different psychiatric pathologies, demonstrating a good response^26,27^. In our research, we used the Brazilian version of FAB. This battery consists of six subtests: Similarities; Lexical Fluency; Motor Series; Conflicting Instruction; Go-No Go Task; Prehension Behavior. The maximum score for each subtest is three points (with higher scores indicating better performance), and the total score of the test is calculated by adding the scores of the six subtests (maximum score = 18). Any performance score between 15 and 18 indicates a frontal lobe without disabilities, between 11 and 14 is considered a moderate impairment, and below 10 is considered a severe impairment. These score cutoffs were validated in a Portuguese population^25^.

Regarding functionality, several tools to assess the functionality of bipolar patients have been used until now, with the FAST test being one of them. This test was validated in different populations and at different ages, showing consistent results in BD patients^28,29^. The FAST scores are evaluated through six functional domains: Autonomy; Occupational Functioning; Cognitive Functioning; Financial Issues; Interpersonal Relationships and Leisure Time, where higher scores indicate worse performance, and the total score of the test is calculated by adding the scores of the six subtests (maximum score = 72). Four categories were established in the FAST scale of functional impairment cutoffs: between zero and 11 (no impairment), between 12 and 20 (mild impairment); between 21 and 40 (moderate impairment), and scores above 40 indicate (severe impairment). However, patients are not static in a category after an intervention; either pharmacological or psychological patients can interchange through categories^22,24^. In this research, we tried to establish the degree of functional impairment through FAST and executive functions through FAB tests by analyzing a group of BD I/II patients in their euthymic phase compared with a healthy control group.

### In vivo studies

#### Erythrocyte and plasma preparation

To analyze the antioxidant enzymes (GSH-Px, SOD, and CAT) and TBA-RS, 10 mL of peripheral blood was taken from each individual by venipuncture in a vacuum tube with heparin. Blood samples were centrifuged at 1,000-x g for 10 min; plasma was then removed by aspiration and frozen at -80 °C until use in TBA-RS assays. The erythrocytes were separated from the plasma, and after that, the erythrocytes were washed 3 times with cold saline (4–8 °C) (0.153 mol/L sodium chloride). Subsequently, the erythrocytes were lysed by adding 1 mL of distilled water to 100 µL of washed erythrocytes, and then they were frozen at -80 °C until the determination of the antioxidant enzyme activities. Posteriorly, they were frozen and thawed 3 times and centrifuged at 13,500 × g for 10 min. To determine the activity of antioxidant enzymes, the supernatant was diluted to contain approximately 0.5 mg/mL protein. All samples were assessed in duplicate.

#### Thiobarbituric acid reactive substances assay (TBA-RS)

The method used to assay the TBA-RS levels was described by Ohkawa et al. (1979)^30^. The methodology measures malondialdehyde (MDA), a product of lipoperoxidation, caused mainly by hydroxyl free radicals. The plasma was mixed with 20% trichloroacetic acid and 0.8% thiobarbituric acid and heated in a boiling water bath for 60 min. A calibration curve was used 1,1,3,3-tetramethoxypropane as a precursor to MDA, and each curve point will be subjected to the same treatment as the supernatants. These complexes are stained, and their concentration can be determined spectrophotometrically at 535 nm. The results were expressed as nanomoles of MDA formed per milligram of protein.

#### Superoxide dismutase assay (SOD)

The method used to assay the SOD activity was described by Marklund, (1985)^31^. The method is based on the capacity of pyrogallol to autoxidize, a process highly dependent on superoxide (O_2_-), which is a substrate for SOD. Briefly, to 15 mL of each sample, 215 mL of a mixture containing 50 mM Tris buffer, pH 8.2, 1 mM EDTA and 30 mM CAT was added. Subsequently, 20 mL of pyrogallol was added, and the absorbance was immediately recorded every 30 s for 3 min at 420 nm using a UV–visible Shimadzu spectrophotometer. The inhibition of autoxidation of pyrogallol occurs in the presence of SOD, whose activity can be indirectly assayed spectrophotometrically. A calibration curve was generated with purified SOD as a reference to calculate the activity of SOD present in the samples. One SOD unit is defined as the amount of SOD necessary to inhibit 50% of pyrogallol autoxidation, and the specific activity is reported as SOD units/mg protein.

#### Catalase assay (CAT)

The method used to assay the CAT activity was described by Aebi, (1984)^32^. The method is based on the using a UV–visible Shimadzu spectrophotometer. The method used was based on the disappearance of H_2_O_2_ at 240 nm in a reaction medium containing 20 mM H_2_O_2_, 0.1% Triton X-100, 10 mM potassium phosphate buffer, pH 7.0, and 0.1–0.3 mg protein/mL. One CAT unit was defined as 1 mmol of H_2_O_2_ consumed per minute, and the specific activity was calculated as CAT units/mg protein.

#### Glutathione peroxidase assay (GSH-Px)

The method used to assay the GSH-Px activity was described by Wendel, (1981)^33^. The method is based on the using Using tert-butyl-hydroperoxide as a substrate. NADPH disappearance was monitored at 340 nm using a UV–visible Shimadzu spectrophotometer. The medium contained 2 mM GSH, 0.15 U/mL GSH reductase, 0.4 mM azide, 0.5 mM tertbutyl-hydroperoxide and 0.1 mM NADPH. One GSH-Px unit was defined as 1 mM NADPH consumed per minute, and the specific activity is presented as GSH-Px units/mg protein.

### Statistical analysis

Demographic and clinical variables were analyzed using descriptive statistics, including (mean) and (standard deviation) for quantitative variables and absolute frequency (n) and relatives (%), for qualitative variables with a confidence interval of 95% in both cases. For the qualitative nominal and ordinal data, we used the chi-square test (χ2) of Pearson, and for two or more groups, we used Fisher's exact test. Parametric and nonparametric tests were used for the analysis of qualitative variables. The assumption of normality and homoscedasticity of each variable was analyzed with the Kolmogorov–Smirnov normality test and Levene's test, respectively. For comparisons of parametric variables between two groups, Student’s *t-*test was used, and for more than two groups, Tukey's test of analysis of variance (ANOVA) was used. To compare nonparametric variables between two and three independent samples, Mann–Whitney tests and Kruskal–Wallis tests were used. Dunn's post hoc test was performed for peer comparisons if the main effects were significant. For association analyses, Pearson correlation was used to test quantitative variables, and Spearman correlation was used for nonquantitative variables. The SPSS software program (SPSS Inc., Chicago, USA) was used. To calculate the statistical power analyses, we used the program G*Power 3.1. Statistical significance was set at *p* < 0.05 for all tests, or a level of significance of 5% was adopted to reject the null hypotheses.

### Research ethics commitee

This study was approved by the Research Ethics Committee of Universidade da Região de Joinville—UNIVILLE (protocol number 655.037) and followed the ethical rules of the Helsinki Declaration of 1975. All participants provided written informed consent before entering the study.

## Results

### Demographic, clinical and pharmacological characteristics

Our research group, in a previously published article, Cyrino et al. (2021)^34^, analyzed the effects of BD in euthymic patients in two subgroups of patients (≤ 3 years and more than ≥ 10 years of the disease) and we measured the cognitive functions and functionality using the FAB and FAST tests respectively. Part of our previous results, were used in this article, aiming to correlate with the level of oxidative stress, and to better understand the neuroinflammation and neuroprogression process in BD.

We evaluated the demographic and clinical characteristics of the different groups studied. Fifty patients with BD were divided into two groups of euthymic patients (≤ 3 and ≥ 10 years of the disease) with 25 patients each. Thirty-six euthymic patients (72%) were female. There was a significant difference in the mean age as in the mean years of education between the healthy control group, and both euthymic patient groups (≤ 3 years and ≥ 10 years of the disease)**.** There was nonsignificant difference between the groups in gender, occupational status, or marital status as shown in Table 1.

**Table 1:** Sociodemographic characteristics of the sample.

	Healthy controls n = 25	Bipolar patients ≤ 3 years of the disease n = 25	Bipolar patients ≥ 10 years of the disease n = 25	*p* value
Age, years^b^	35.0 (± 9.96)	34.9 (± 10.04)	47.4 (± 8.21)	*p* < 0 0.01^b^
**Gender, n**				*p* = 0.77^a^
Male	9	7	7	
Female	16	18	18	
**Marital status n (%)**				*p* = 0.08^d^
Married	12 (48)	9 (36)	15 (64)	
Divorced	1 (4)	2 (8)	5 (20)	
Widowed	1 (4)	0 (0)	1 (4)	
Single	11 (44)	14 (56)	4 (12)	
**Education n (%)**				*p* = 0 0.13^d^
Illiterate	-	-	-	
Up to primary school	0 (0)	3 (12)	4 (16)	
Up to high school	10 (40)	10 (40)	12 (48)	
Graduate	12 (48)	12 (48)	9 (36)	
Postgraduate	3 (12)	0 (0)	0 (0)	
**Years of education**^**b**^	14.7 (± 2.18)	13.8 (± 2.70)	12.4 (± 2.77)	*p* < 0.01^e^
**Work situation n (%)**				*p* = 0.17^d^
Employed	23 (92)	18 (72)	13 (52)	
Unemployed	2 (8)	6 (24)	10 (40)	
Medical benefits	0 (0)	1 (4)	0 (0)	
Invalidity	0 (0)	0 (0)	2 (8)	

^a^χ2, ^b^Mean (± SD), ^c^One-way ANOVA followed by Dunn´s post hoc, ^d^Fisher's exact test e Kruskal Wallis.

Regarding pharmacologic treatment, our results showed that 10 (20%) of the patients were on monotherapy. Among the patients on polypharmacy, 18 (36%), 16 (32%), and 6 (12%) of the patients received 2, 3, and 4 psychotropic medications, respectively, as shown in Table 2.

**Table 2:** Clinical and pharmacological characteristics of the sample.

	Healthy controls n = 25	Bipolar patients ≤ 3 years of the disease n = 25	Bipolar patients ≥ 10 years of the disease n = 25	*p* value
Illness duration (years)^a^	N/A	2.52 (± 0.65)	15.64 (± 6.81)	*p* < 0.001^d^
Age of onset (years)^a^	N/A	22.1 (± 7.01)	25.1 (± 6.17)	*p* = 0.62^d^
HAMD-17 total score^a^	4.32 (± 2.49)	4.10 (± 2.02)	3.71 (± 1.46)	*p* = 0.53^b^
YMRS total score^a^	0.56 (± 0.86)	0.88 (± 1.01)	1.28 (± 1.13)	*p* = 0.08^c^
FAST score, median (IQR)	9 (7)	22 (10)	23 (20)	*p* < 0.001^c^
FAB score, median (IQR)	16 (3)	14 (4.5)	14 (3,5)	*p* < 0.001^b^
Hospitalizations n (%)	N/A	12 (48)	8 (32)	
Duration hospitalizations (day)^a^	N/A	13.4 (± 24.3)	13.0 (± 36.6)	*p* = 0.90^d^
Suicide attempts n	N/A	18	40	
Family history of affective disorders n (%)	N/A	10 (40)	13 (52)	
Psychoeducation Yes, n (%)	N/A	21 (84)	20 (80)	
**Treatment n (%)**				
Lithium	N/A	13 (52)	15 (60)	
Other mood stabilizers	N/A	11 (44)	13 (52)	
Atypical antipsychotics	N/A	8 (32)	12 (48)	
Typical antipsychotics	N/A	2 (8)	0 (0)	
Antidepressants	N/A	7 (28)	7 (28)	
Benzodiazepines	N/A	2 (8)	7 (28)	

HAMD-17 = Hamilton Depression Rating Scale; YMRS = Young Mania Rating Scale; FAST = Functioning Assessment Short Test; FAB = Frontal Assessment.

Battery; N/A = not available; IQR = interquartile range a Mean (±SD) b t test c Mann-Whitney.

### Evaluation of the levels of oxidative stress mediators in erythrocytes

Evaluation of the activities of SOD, GSH-Px, and CAT in erythrocytes and TBA-RS levels in the plasma of the sample groups are presented in Table 3 as the mean and standard deviation. Regarding the parameters of oxidative stress, bipolar patients showed increased oxidative damage to lipids, with an increase in TBA-RS levels in erythrocytes in both groups (≤ 3 years and ≥ 10 years since the onset of the disease), compared to the control group [F (2,72) = 21.6]. Regarding antioxidant defense, patients had increased CAT activity in erythrocytes in both groups (≤ 3 years and ≥ 10 years since disease onset) compared to the control group [F (2,72) = 17.13]. However, no changes were shown in the activity of the SOD and GSH-Px enzymes in any of the groups.

**Table 3:** Comparison of Oxidative Stress Parameters between the healthy control patients and the Euthymic bipolar patients with ≤ 3 and ≥ 10 years of the disease.

	Healthy control patients n = 25	Euthymic patients BD ≤ 3 yearsn = 25	Euthymic patients BD ≥ 10 years n = 25	*p* – Value
Oxidative stress parameters				
SOD (units/mg protein) means (± SD)	5.35 (± 0.26)	5.32 (± 0.39)	5.50 (± 0.42)	0.17^a^
GSH-Px (units/mg protein) means (± SD)	5.80 (± 0.99)	5.65 (± 0.95)	6.18 (± 1.48)	0.25^a^
CAT (units/mg protein) means (± SD)	3.47 (± 0.77)	8.10 (± 4.18)	9.41 (± 4.95)	**0.001^b^
TBA-RS (nmol MDA/mg protein ) means (± SD)	1.62 (± 0.28)	3.18 (± 1.17)	3.01 (± 1.03)	**0.001^a^

Means ± standard deviation (SD). The normality of each variable was analyzed using the Kolmogorov–Smirnov normality test. ^a^To compare parametric variables between the three independent groups, two-way analysis of variance (ANOVA) followed by Tukey's test was used. ^b^To compare nonparametric variables between the three independent samples, the Kruskal–Wallis test was used. Dunn's post hoc test was performed for pairwise comparisons if the main effects were significant. Statistical significance was set at ** *p* < 0.001 for all tests.

### Functional and neurocognitive performance

#### FAST and FAB scores in healthy controls and euthymic patients with ≤ 3 years and ≥ 10 years of the disease

After performing the FAST test, the results showed a significant difference between the healthy control group and the groups with ≤ 3 and ≥ 10 years of the disease**.** All effect sizes (f2) were in the same direction, suggesting worse performance in the euthymic patient groups. However, in the FAB test we observed a significant difference between the groups ≤ 3 and ≥ 10 years of the disease**,** but no significant difference between the groups with ≤ 3 years of the disease and the healthy control group, as shown in Table 4.

**Table 4:** Mean total of FAST and FAB scores in healthy controls and euthymic patients with ≤ 3 and ≥ 10 years of the disease.

	Healthy control patients n = 25	Euthymic patients BD ≤ 3 years of disease n = 25	Euthymic patients BD ≥ 10 years of disease n = 25	*p* Value	f_2_
Fast Meaans (± SD)^a^	9.80 (± 5.94)	20.63(± 8.21)	27.80 (± 12.50)	< 0.001**	0.7960
FAB Meaans (± SD)^b^	15.84(± 1.55)	14.64(± 2.48)	12.44(± 2.78)	< 0.001**	0.6042

Means ± standard deviation (SD). FAB = Frontal Assessment Battery FAST = Functioning Assessment Short Test. The normality of each variable was analyzed using the Kolmogorov–Smirnov normality test.

^a^To compare nonparametric variables between the three independent samples, the Kruskal–Wallis test was used. Dunn's post hoc test was performed for pairwise comparisons if the main effects were significant.

^b^To compare parametric variables between the three independent groups, two-way analysis of variance (ANOVA) followed by Tukey's test was used. Statistical significance was set at ** p < 0.001 for all tests. *f*^*2*^ = the overall Cohen's effect size. Image origin: Cyrino et al., (2021). Assessment of different domain impairments in Cognitive Functions and Functionalities found in Euthymic Patients with Bipolar Disorder I / II—during the early and late phases of the disease, using the FAB and FAST tests. International Journal of Advanced Engineering Research and Science (IJAERS). 8, 401–433.

#### Correlation between FAST and FAB test scores in euthymic patients

The correlation between FAB and FAST tests was analyzed through the scores of both tests in euthymic patients using the Spearman correlation coefficient after assessing normality using the Shapiro–Wilk test. Although only a few samples of euthymic patients in each group were used (n = 25), it was possible to observe a negative correlation present, with a low intensity (R^2^ = -0.350) in patients with ≤ 3 years of the disease and with moderate intensity (R2 = -0.437) in patients with ≥ 10 years of the disease.

#### Correlation between FAB test and the levels of TBA-RS in euthymic patients and FAB test scores in euthymic patients

The correlations between the plasma levels of TBA-RS and FAB test scores and the correlation of TBA-RS plasma levels and FAST test scores in euthymic bipolar patients were analyzed using the Pearson and Spearman correlation coefficients, respectively, after evaluating the normality test using the Shapiro–Wilk test.

#### Category scores of the FAST test in healthy controls and euthymic patients

There was no statistical significance between the ≤ 3 and ≥ 10 years of the disease groups, but there was a significant difference in all distinct domains of FAST test between the two-euthymic patients groups when comparing them with the healthy control group. All effect sizes (f_2_) were in the same direction, showing a decrease mainly in occupational, autonomy, cognitive and interpersonal domains, as shown in Table 5.

**Table 5:** Results of FAST substests in euthymic patients with ≤ 3 years and ≥ 10 years of the disease.

	Healthy control n = 25 (a)	Euthymic patients BD ≤ 3 years n = 25 (b)	Euthymic patients BD ≥ 10 years n = 25 (c)	*p *value	POS-HOC analysis	f_2_
Fast total score	9.80 (± 5.94)	20.63 (± 8.21)	27.80 (± 12.50)	*p* < 0.001**^a^	a < b a < c b ≈ c	0.796
FAST subtests						
*1. Autonomy*	1.20 (± 1.76)	3.32 (± 2.54)	3.16 (± 2.71)	*p* < 0.001**^a^	a < b a < c b ≈ c	0.405
*2. Occupational functioning*	0.96 (± 1.10)	5.48 (± 4.02)	5.16 (± 4.53)	*p* < 0.001**^a^	a < b a < c b ≈ c	0.579
*3. Cognitive functioning*	4.00 (± 2.25)	7.92 (± 3.36)	7.08 (± 3.95)	*p* < 0.001**^a^	a < b a < c b ≈ c	0.515
*4. Financial issues*	1.32 (± 1.49)	2.96 (± 2.20)	2.48 (± 2.00)	*p* < 0.01**^a^	a < b a < c b ≈ c	0.357
*5. Interpersonal relationship*	1.24 (± 1.56)	5.44 (± 4.47)	4.8 (± 3.76)	*p* < 0.001**^a^	a < b a < c b ≈ c	0.529
*6. Leisure time*	1.20 (± 1.22)	2.62 (± 2.39)	2.80 (± 2.19)	*p* < 0.04*^a^	a < b a < c b ≈ c	0.354

Means ± standard deviation (SD). FAST = Functionality Assessment Short Test. n.s. = no significant ^a^Kruskal–Wallis followed by Dunn´s post hoc to FAST test. [*] indicate FAST scores significantly different between groups. * *p* < 0.05 ** *p* < 0.001. f_2_ = the overall Cohen’s effect size.

This image was obtained with permission (IJAERS). Image origin: Cyrino et al., (2021). Assessment of different domain impairments in Cognitive Functions and Functionalities found in Euthymic Patients with Bipolar Disorder I/II—during the early and late phases of the disease, using the FAB and FAST tests. International Journal of Advanced Engineering Research and Science (IJAERS). 8, 401–433.

#### Categories score of FAB test in healthy controls and euthymic patients

There was no statistical significance between the ≤ 3 and ≥ 10 years of the disease groups, but there was a significant difference in four distinct domains between the group with ≥ 10 years of the disease and the healthy control group.

All effect sizes (f2) were in the same direction, suggesting worse performance in the euthymic patient groups than in the healthy control group, as shown in Table 6.

**Table 6:** Results of FAB subtests in Euthymic Patients with ≤ 3 years and ≥ 10 years of the disease.

	Healthy control n = 25 (a)	Euthymic patients BD ≤ 3 years n = 25 (b)	Euthymic patients BD ≥ 10 years n = 25 (c)	*p *value	POS-HOC analysis	f_2_
FAB total score	15.84 (± 1.55)	14.64 (± 2.48)	12.44 (± 2.78)	*p* < 0.001^∗∗a^	a ≈ b b > c a > c	0.6042
FAB subtests						
*1. Similarities (conceptualization)*	1.88 (± 0.88)	1.52 (± 1.15)	1.28 (± 1.02)	*p* < 0.03*^a^	a ≈ b b ≈ c a > c	0.240
*2. Lexical fluency (mental flexibility)*	2.68 (± 0.47)	2.48 (± 0.58)	2.56 (± 0.65)	*p* > 0.05^a^	n.s	0.143
*3. Motor series (motor programming)*	2.92 (± 0.27)	2.84 (± 0.47)	2.48 (± 0.87)	*p* < 0.02*^a^	a ≈ b b ≈ c a > c	0.323
*4. Conflicting instruction (sensitivity to interference)*	2.92 (± 0.27)	2.56 (± 0.77)	2.36 (± 0.95)	*p* < 0.02*^a^	a ≈ b b ≈ c a > c	0.331
*5. Go-no go task (inhibitory control)*	2.32 (± 1.14)	1.52 (± 1.50)	1.60 (± 1.41)	*p* < 0.05*^a^	a ≈ b b ≈ c a > c	0.299
*6. Prehension behaviour (environmental autonomy)*	2.96 (± 0.20)	2.84 (± 0.47)	2.76 (± 0.72)	*p* > 0.05^a^	n.s	0.274

Means ± standard deviation (SD). FAB = Frontal Assessment Battery. n.s. = no significant ^a^Kruskal–Wallis followed by Dunn´s post hoc to FAB test. [*] indicate FAB scores significantly different between groups. * *p* < 0.05 ** *p* < 0.001. f_2_ = the overall Cohen’s effect size.

This image was obtained with permission (IJAERS). Image origin: Cyrino et al., (2021). Assessment of different domain impairments in Cognitive Functions and Functionalities found in Euthymic Patients with Bipolar Disorder I / II - during the early and late phases of the disease, using the FAB and FAST tests. International Journal of Advanced Engineering Research and Science (IJAERS). 8, 401-433.

## Discussion

Our results were quite consistent with the neuroprogression theory. We observed that biomarkers related to oxidative stress (SOD, CAT, GSH-Px, and TBA-RS) promoted impairments in frontal cognitive functions and functional alterations, reinforcing the results of other similar studies. Our group focused on reducing any biases that could interfere with the measurement of these inflammatory biomarkers and the results collected by using very strict inclusion and exclusion criteria for bipolar patients, as described previously. The patients were in the euthymic phase for at least six months. In addition, patients were not allowed to use any medications other than those that were prescribed for their psychiatric condition, and they should have been using them for at least four weeks. However, all 50 bipolar patients participating in this research were medicated with one, two, or more psychotropic drugs of different classes (lithium, valproic acid, lamotrigine, and second-generation antipsychotics).

There is some research showing that individuals with BD in the different evolutionary stages of the disease (early and late) and different phases (depressive, manic, and euthymic) seem to present alterations in the different groups of biomarkers in the peripheral blood^35–37^.

In our study, we did not observe changes in SOD and GSH-Px activities. However, we observed significant increases in CAT activity and plasma TBA-RS levels, both in the ≤ 3 years and ≥ 10 years groups with the disease being higher in euthymic patients than in healthy controls, see Table 3. These SOD and GSH-Px activities are in agreement with several studies carried out in which SOD and GSH-Px activities are related to mood episodes, where they are increased during manic and depressive phases but not during euthymia. In addition, these enzymes in unmedicated manic patients returned to normal levels after treatment with mood stabilizers, showing that the use of mood stabilizers alters oxidative stress and stabilizes the activity of antioxidant enzymes. These studies are in line with our results, where we did not observe any change in any patients in the medicated bipolar group in the euthymic phase or control group^36,38,39^.

However, regarding CAT activity enzyme and TBA-RS levels, we found the opposite result. Although the patients were euthymic and medicated, the CAT activity enzyme and TBA-RS levels were increased in both groups. Thus, even patients who had been diagnosed recently (≤ 3 years of the disease) had a strong increase in TBA-RS levels and CAT activity, demonstrating that the oxidative stress that started in the initial phases was maintained^40,41^. Our results have many similarities when we compare the results of a large meta-analysis carried out by Jiménez‐Fernández, 2020^42^. They analyzed 44 studies, which included 1979 bipolar patients and 1788 healthy control patients, evaluating the following oxidative stress markers: SOD, CAT, GSH-Px enzymes, and TBA-RS levels during different phases of the disease (mania, depression, and euthymia), and separated them into subgroups (use and nonuse of mood-stabilizing drugs). The results found by the authors partially reflect the results of three previous meta-analyses^43–45^. Thus, when Jiménez-Fernández et al. (2020)^42^ compared healthy controls with euthymic medicated bipolar patients in their meta-analysis, they had the following results: CAT activity scores **(*p* < 0.02),** TBA-RS levels (*p* < 0.0001), and without differences relating to SOD activity (*p* = 0.10), and GSH-Px activity (*p* = 0.40). These results are the same as those observed in our research, even when we divided the patients into two groups (≤ 3 years and ≥ 10 years of the disease), with an increase in CAT activity (≤ 3 years *p* < 0.0001; ≥ 10 years *p* < 0.0001) and TBA-RS levels (≤ 3 years *p* < 0.0001; ≥ 10 years* p* < 0.0001) and with no significant difference related to SOD activity (≤ 3 years *p* = 0,9; 10 years *p* = 0.3) and GSH-Px activity (≤ 3 years *p* = 0.8; ≥ 10 years *p* = 0.3) see Table 3.

All of these findings offer an attractive explanation for the cognitive and behavioral impairment, based on our results of the FAB and FAST tests, reflecting the morphological and neuronal alterations produced in the prefrontal cortex, even during euthymia^46^. The prefrontal cortex is a heterogeneous region comprising several specialized subregions, where the three main regions are the orbitofrontal, ventromedial, and dorsolateral regions. The prefrontal cortex communicates with the entire brain, receiving and sending projections of all kinds and integrating with different areas and systems. Each region has specific functions, and any neurofunctional alteration causes harm with behavioral and clinical changes. Impairments in executive functions, impulsivity, and apathy, for example, are characteristics of dysfunctions in the frontal-subcortical circuit present in neuropsychiatric disorders, such as BD. Recently, numerous researchers^47–49^, have sought to relate the six domains present in the FAB test, as shown in Table 6 and Fig. 2. The final impact of neurocognitive impairment is the loss of functionality of these patients, especially with the inability to perform daily activities. Cognitive failures, general work, and difficulties in interpersonal relationships are the main problems presented by these patients^50^, as shown in Table 5 and Fig. 3. Our results showed that functional alterations were present since the beginning of the disease, emphasizing the core of our study, as shown in Tables 5 and 6; Figs. 2; 3.

As mentioned above, in BD, a likely hypothesis is that the imbalance between pro- and anti-inflammatory factors produces a reduction in neurotrophins and increased oxidative stress (ROS), which occurs due to mitochondrial dysfunction involving disruption of the ETC, which is considered the main cause of chronic oxidative stress in BD. All this process develops neuroinflammation, and it plays an important role in the pathophysiology of BD, producing a continuous pathophysiological cycle of aggravation of the disease and leading to a metabolic disturbance in neurons and glial cells^6,51^**.** Physiologically, ROS act as essential second messengers in innate and adaptive immunity, stimulating the generation of proinflammatory cytokines (including IL-1β, IL-6, TNF-α, and interferons) during the immune response to control pathogens and repair tissue damage**.** However, the oxidative stress process constantly produces ROS, and when in excess, they can cause the oxidation of biological molecules. Of all the related ROS, O_2_•- is the main product formed by ETC complexes I and III in mitochondria, and therefore, production is increased in metabolically active neurons. Normally, O_2_ acts as an electron-receiving molecule in the ETC and is reduced to H_2_O (water). However, under certain conditions, O_2_ accepts only one electron, forming O_2_•-. It has little reactivity in an aqueous medium; however, when in an acidic medium, it can form H_2_O_2_. H_2_O_2_ is not strongly reactive, but it can be toxic due to its long half-life and its ability to cross lipid membranes, impairing membrane permeability**.** In addition, by reacting with the erythrocyte membrane and iron-bound proteins, they cause damage to molecules through the generation of new ROS, such as OH•^52^**. **OH• is the most reactive biological species and can damage, for example, ETC I, II, and III complexes, decreasing their activities. It can also lead to malfunction of the Fe-S electron center complex in the tricarboxylic acid cycle and affect mtDNA, leading to more oxidative stress and resulting in a reduction in mitochondrial energy production^53^**.** Various enzymatic and nonenzymatic antioxidant defense systems can reduce the damage caused by ROS production. However, most ROS are neutralized by endogenous enzymatic antioxidants, which are best known as SOD, CAT, and GSH-Px enzymes^17^. The SOD enzyme acts as the main protective enzyme against oxidative stress and DNA damage in the mitochondria, catalyzing the dismutation of O_2_•- into H_2_O_2_ and O_2_**.** In turn, the generated H_2_O_2_ is then partially eliminated by CAT and GSH-Px enzymes, which convert H_2_O_2_ to H_2_O and O_2._ Thus, both the GSH-Px enzyme, which acts inside the mitochondria but has low efficiency, and the CAT enzyme, which acts in the cytoplasm and is present in almost all cells, are highly efficient. Both can inhibit lipid peroxidation, preventing any loss of membrane functions^19,54^**.**

The H_2_O_2,_ when isolated, is practically innocuous, and in low quantities, it tends to regulate some physiological processes. However, when in high concentrations, it binds to Fe +  + , as it is more available in the body, a reaction called (Fenton reaction) occurs, which can generate OH• radicals inside the cell, which are the most harmful of all**.** Therefore, CAT and GSH-Px activity enzymes are critical for limiting the damage induced by the concentration of H_2_O_2_ in cells. Furthermore, the CAT enzyme continuously searches for H_2_O_2_ molecules, which can break down millions of them per second and are located mainly in the peroxisomes of mammalian cells^35,36^**.**

Thus, the increase in CAT activity enzyme and the TBA-RS levels in euthymic bipolar patients, both with patients ≤ 3 years and with more than ≥ 10 years of the disease compared to healthy controls, suggests an increased but relatively stable biochemical condition during euthymia and no important differences with the time of illness. These persistent biochemical changes in euthymia may be a sign of the continuation of the illness despite the absence of clinical symptoms. In other words, neuroinflammation persists in euthymia, and the neuroprogression of the disease continues to evolve, although the patients do not present clinical symptoms. Thus, Selek et al. (2015)^55^, studying the CAT activity enzyme, observed a correlation with the time of disease, which may be a sign of an increase in compensatory mechanisms due to the chronicity of the disease. For the significantly increased levels of TBA-RS, as found in our work, both in bipolar patients with ≤ 3 years and ≥ 10 years of the disease, it seems highly plausible to think that chronic stressful stimuli significantly alter their levels (although the maintenance of these increased levels remains unclear). This chronic increase in TBA-Rs compromises the biochemical integrity of the cell membranes of neurons in the prefrontal cortex, producing a lipid peroxidation process, altering plasticity, regenerative capacity, and dendritic architecture with shrinkage and decreased neuronal connectivity, leading to a decrease in BDNF and thus disturbing normal synaptic neurotransmission by oxidation of glutamate NMDA receptors, leading to attenuation of long-term potentials and synaptic neurotransmission^46^**.** These neurofunctional changes can start an autovicious cycle in which various systems and mechanisms are exacerbated and accelerate cell damage, resulting in progressive structural brain changes and impairment of frontal cognitive functions. All these changes seem to contribute to BD neuroprogression^6,56^**.**

Therefore, we can hypothesize that bipolar patients have a neuroinflammatory process in the brain since the first episodes of mania and depression, and the euthymic state in BD is not synonymous with the recovery of the patient's functionality. Both ideas are critical for understanding the mechanism and evolution related to BD. It was very well described by Gitlin et al. (1995)^57^**,** who demonstrated that despite pharmacological and psychotherapeutic treatment, 73% of patients relapsed with depression and mania many times within five years. Even for those who did not relapse, alterations in their psychosocial functioning were observed, mainly in the occupational and interpersonal areas, generating a poor prognosis for the disease^58,59^. However, the most recently observed hypothesis related to this phenomenon would be the cognitive deficits resulting from the chronicity of the clinical course and the persistent subsyndromal symptoms that remain present**.** Furthermore, research with patients shortly after the first manic episode showed that functional impairments were already present in up to 70% of them^60^**.** Thus, functional impairment, in the most different aspects, was not significantly different in patients during their first episode than in those with multiple episodes. However, there are still few studies that compare the profile of the neurocognitive and functional performance of BD patients, especially in the euthymic phase, and even more, studies that differentiate and correlate the degree of biochemical alterations with cognitive-functional aspects among patients who more recently developed the disorder (early stage of the disease) with those longer disease duration (late stage of the disease) are necessary.

This chronic and slow inflammation causes patients to seek treatment long after the first signs of the disease appear, which can hinder a specialist from making the correct diagnosis and admitting proper treatment and can vary from five to ten years. Thus, the early use of medications would be beneficial in the patient's clinical evolution, and they are effective in 80% of BD cases, having a neuroprotective effect. Thus, treating the disease immediately after the first episode of depression or mania preserves the body's ability to recover and maintain these patients in euthymia^61^**.** However, it was found that 40% of these patients maintained the disease under control by taking three or more medications. After the tenth crisis, BD gains autonomy and can independently trigger previous stressful conditions (a process called kindling). Furthermore, it is important to emphasize that even lithium loses its effectiveness after the tenth outbreak, causing more frequent and prolonged episodes, with the intervals between them becoming shorter^48^.

**Figure 1:**
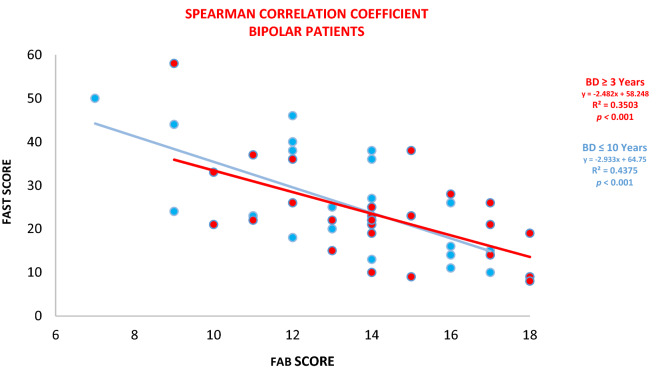
Correlation between FAST and FAB tests scores between BD patients ≤ 3 years (in red) and ≥ 10 years of the disease (in blue) using the Spearman Correlation Coefficient. This image was obtained with permission (IJAERS). Image origin: Cyrino et al., (2021). Assessment of different domain impairments in Cognitive Functions and Functionalities found in Euthymic Patients with Bipolar Disorder I / II—during the early and late phases of the disease, using the FAB and FAST tests. International Journal of Advanced Engineering Research and Science (IJAERS). 8, 401–433.

**Figure 2:**
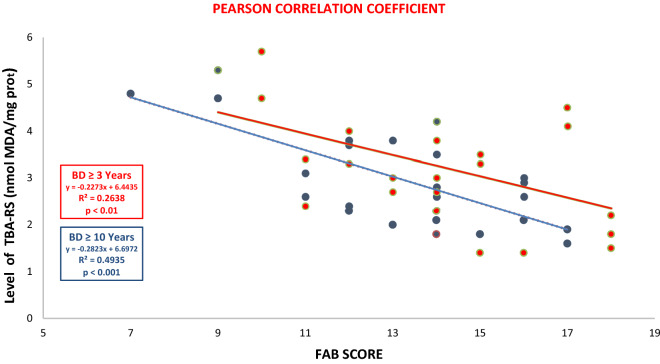
Correlation between Levels of TBA-RS and FAB test scores between BD patients ≤ 3 years (in red) and ≥ 10 years of the disease (in blue) using the Pearson correlation coefficient.

**Figure 3:**
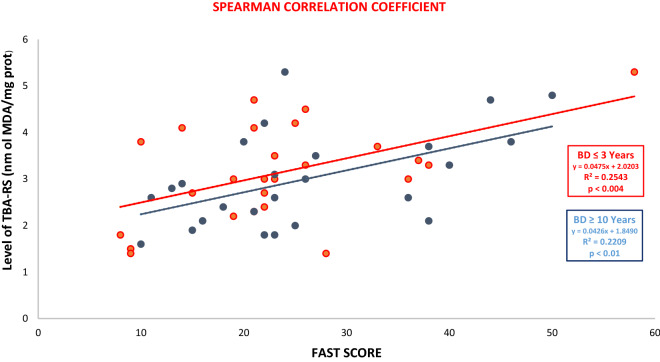
Correlation between Levels of TBA-RS and FAST test scores between BD patients ≤ 3 years (in red) and ≥ 10 years of the disease (in blue) using the Spearman correlation coefficient.

These results reinforced the previous hypothesis, which is the correlation between the variables FAB and FAST, and demonstrated that euthymic patients who have lower scores on the FAB (decreased frontal activities) had higher scores on the FAST (with greater loss of functionality), as seen in Figs. 1, 2, and 3. Since the results of the correlation coefficient (R^2^) represent a low to moderate correlation, the FAB variable alone cannot explain the total FAST variability. However, the sample results provide very significant statistical evidence between FAB and FAST**.** It is significant to emphasize that the recovery is partial, contrary to what was initially thought. Although most patients present recovery relating to clinical symptoms, functional recovery rates range between 30 and 40%^62^**.**

Thus, the concept of neuroprogression explains the clinical symptoms well, but it is still not sufficient to know whether all these changes are the cause or a consequence of the disease. However, it seems prudent that from a clinical perspective, we should think about starting treatment as soon as possible and keep it for an extended period. Evidence, more consistently, will require the monitoring of patients for several years with laboratory tests, imaging, and neuropsychological tests from time to time to assess the evolution of the problem. Although it is far from being proven yet, this proposal is leading to further research for more specific and efficient therapies and the development of better strategies that allow us to identify any patients at risk of developing these pathologies. Thus, the treatment of BD is based on the management of acute episodes (seeking to lead a patient in mania or depression to the remission of symptoms—euthymia). On the other hand, in chronic treatment, the aim is to maintain the euthymic state to prevent the occurrence of new episodes, reduce subsyndromal symptoms and increase the functionality of patients.

Therefore, although our results corroborate other similar studies and support the hypothesis that oxidative stress is important in BD, we can point to some limitations of our results. Despite implementing precautions to avoid any biases as much as possible, we must emphasize that the small number of participants in each subgroup can produce an analysis that must be interpreted within its limitations. Although it was not the objective of our study, we did not consider genetic factors, since depression and BD are related to different pro-oxidant and antioxidant genetic polymorphisms that increase susceptibility to BD. Another point is that our work is a cross-sectional analysis, and therefore, we cannot compare different types of treatment (mood stabilizers) and their possible effects, although the literature demonstrates that all these agents have antioxidant properties. Due to these discrepant results, these enzymes cannot yet be used as BD biomarkers, but they may be useful to assess disease stages.

This ultimately suggests that when systemic toxicity is present during the mania and depressive phases, they may improve the conditions of these cells when controlled, reinforcing the idea that the different modalities of treatments lead the patient to a better quality of life. In light of this interpretation, the goal of treatment is no longer just the remission of clinical symptoms but rather seeks to prevent relapses, thus helping to maintain cognitive and functional capacity over time. This conclusion becomes one of the reference points in this study because regardless of the approach used during the treatment (pharmacological, psychotherapy, and psychoeducation), the need for an increasingly early diagnosis and intervention becomes imminent so that different forms of therapies can have a neuroprotective effect, which in turn generates an attenuation of clinical symptoms and possible biomarkers, resulting in a more fulfilled life.

## Data Availability

The datasets generated during and/or analyzed during the current study are not publicly available because individual privacy could be compromised but are available from the corresponding author upon reasonable request.
